# Host plant use by spittlebug nymphs in agroecosystems: a field survey supporting targeted vegetation management

**DOI:** 10.1093/jee/toag179

**Published:** 2026-06-27

**Authors:** Maurizio Vincenzo Puzzovivo, Giacomo Santoiemma, Giada Spadavecchia, Nicola Tagarelli, Maria Luisa Vitale, Giuseppe Bari, Rosalia Marroccoli, Vincenzo Verrastro, Vinton Thompson, Daniele Cornara

**Affiliations:** Department of Soil, Plant and Food Science (DiSSPA), University of Bari Aldo Moro, Bari, Italy; International Centre for Advanced Mediterranean Agronomic Studies—Institute of Bari (CIHEAM Bari), Valenzano (Bari), Italy; Department of Agronomy, Food, Natural resources, Animals and Environment (DAFNAE), University of Padua, Legnaro, Italy; Department of Soil, Plant and Food Science (DiSSPA), University of Bari Aldo Moro, Bari, Italy; International Centre for Advanced Mediterranean Agronomic Studies—Institute of Bari (CIHEAM Bari), Valenzano (Bari), Italy; International Centre for Advanced Mediterranean Agronomic Studies—Institute of Bari (CIHEAM Bari), Valenzano (Bari), Italy; Department of Soil, Plant and Food Science (DiSSPA), University of Bari Aldo Moro, Bari, Italy; Department of Soil, Plant and Food Science (DiSSPA), University of Bari Aldo Moro, Bari, Italy; International Centre for Advanced Mediterranean Agronomic Studies—Institute of Bari (CIHEAM Bari), Valenzano (Bari), Italy; American Museum of Natural History, New York, NY, USA; Department of Soil, Plant and Food Science (DiSSPA), University of Bari Aldo Moro, Bari, Italy

**Keywords:** olive agroecosystem, spittlebugs, ground cover management, host plant suitability, insect vector control

## Abstract

Current strategies aimed at containing the spread of the vector-borne bacterium *Xylella fastidiosa* (Wells et al. 1987) include ground cover removal and tillage in order to curb the population of juveniles of spittlebug vectors, such as *Philaenus spumarius* (Linnaeus 1758) (Hemiptera: Aphrophoridae) and *Neophilaenus campestris* (Fallén 1805) (Hemiptera: Aphrophoridae). However, continuous tillage might harm soil, biodiversity, and ecosystem services. Here we present data on host plant use by juveniles of the 2 spittlebug species obtained through a survey carried out in 105 olive orchard plots across Apulia region, South-East Italy. *P. spumarius* juveniles were absent from species such as *Oxalis pes-caprae* (Linnaeus 1753)*, Astragalus hamosus* (Linnaeus), *Mercurialis annua* (Linnaeus 1753)*, Fumaria parviflora* (Lamarck), and *Muscari* spp., while *N. campestris* almost exclusively developed on Poaceae, avoiding most dicots. Host selection was species-specific rather than family-dependent, possibly reflecting differences in xylem chemistry and/or plant architecture. These findings provide empirical support for selective vegetation management: using plant species unsuitable for spittlebug nymphs could reduce vector populations while preserving ground cover benefits, avoiding the ecological shortcomings of indiscriminate removal. However, causal mechanisms remain untested, and the efficacy of this approach in reducing pathogen spread needs experimental validation. Moreover, potential side effects on non-target organisms and ecosystem functions require careful ecological assessment before large-scale implementation.

## Introduction

Plant pathogens transmitted by insect vectors are major drivers of yield reduction and economic losses in agricultural systems worldwide (Savary et al. 2019). In the absence of effective ­curative treatments, disease management primarily relies on suppressing vector populations (Daugherty et al. 2015). However, vector control strategies often produce inconsistent outcomes in limiting pathogen spread and may generate undesirable agronomic, environmental, and human health consequences, highlighting the need for sustainable alternatives within integrated pest management (IPM) frameworks (Avosani et al. 2023, Labadessa et al. 2024). The European containment efforts against the xylem-limited vector-borne bacterium *Xylella fastidiosa* (Wells et al. 1987) (Xanthomonadales: Xanthomonadaceae) provide a clear example of how the management of vector-borne plant pathogens can produce far-reaching consequences, affecting biodiversity, ecosystem services, and human activity, in addition to the direct economic losses caused by disease. In Mediterranean olive agroecosystems, the meadow spittlebug *Philaenus spumarius* (Linnaeus 1758) (Hemiptera: Aphrophoridae) is recognized as the primary driver of olive-to-olive secondary spread of the bacterium, while *Neophilaenus campestris* (Fallén 1805) (Hemiptera: Aphrophoridae) might contribute to pathogen epidemiology, particularly in vineyards (Cornara et al. 2017, Perfetto et al. 2026). Transmission occurs in a persistent, propagative yet non-circulative manner, and persistent systemic infection might be caused even by single inoculation events requiring a relatively short vector-plant contact (Purcell et al. 1979, Hill and Purcell 1995, Purcell 2013). Spittlebugs are xylem sap-feeding insects with a univoltine life cycle, overwintering as eggs. Nymphs emerge in spring and develop on herbaceous vegetation sheltered by a characteristic spittle mass. *P. spumarius* nymphs are primarily associated with dicotyledonous species, whereas *N. campestris* juveniles are mostly observed on monocots (Nickel and Remane 2002). Following adult emergence, individuals disperse to woody hosts before returning to herbaceous vegetation for oviposition (Weaver and King 1954). To reduce vector pressure and bacterium transmission probability, control measures in olive orchards include insecticide applications targeting adults and mechanical removal or tillage of ground cover vegetation during nymphal development (Purcell et al. 1979, Bodino et al. 2019, Sanna et al. 2021). Although widely implemented, the epidemiological effectiveness of these practices remains incompletely quantified, while ecological trade-offs are increasingly reported (Labadessa et al. 2024, Laterza et al. 2026). Repeated tillage and ground cover removal can negatively affect soil structure, reduce organic matter content, and increase erosion rates, particularly in Mediterranean environments prone to drought and desertification (Pimentel and Burgess 2013, García-Ruiz et al. 2015). Vegetation disturbance also reduces habitat complexity and resource availability for beneficial arthropods, including predators, parasitoids, and pollinators (Landis et al. 2000, Bianchi et al. 2006). Loss of ground vegetation has been associated with declines in natural enemy abundance and biological control services, potentially increasing vulnerability to secondary pest outbreaks (Tscharntke et al. 2007). In Mediterranean perennial crops, ground cover contributes significantly to arthropod biodiversity and agroecosystem resilience (Sánchez-Bayo and Wyckhuys 2019). Chemical control strategies also present substantial constraints. Broad-spectrum insecticides may negatively affect non-target organisms, including pollinators and predatory arthropods (Desneux et al. 2007, Pisa et al. 2015), disrupt trophic interactions, and accelerate resistance development in pest populations (Georghiou 1990). Occupational exposure among agricultural workers and environmental contamination of soil and water resources pose additional risks, including neurological disorders, endocrine disruption, and carcinogenicity (Aktar et al. 2009, Mostafalou and Abdollahi 2013, Kim et al. 2017). Importantly, *P. spumarius* and *N. campestris* are native species widely distributed in Mediterranean ecosystems (Cornara et al. 2019). Achieving sustained population suppression at landscape scale through chemical or mechanical approaches alone is therefore inherently challenging. Ecologically informed management strategies that integrate knowledge of vector biology and ecology, including host plant use, may provide more durable solutions. Describing host plant-insect interactions, host suitability refers to the capacity of a botanical species to support insect development and survival, while host preference refers to a behavioral trait of the insect. By contrast, host use describes the realized association between insects and plants in the field (Singer 2000).

One promising approach involves manipulating ground cover composition rather than eliminating it entirely. Adoption of cover crops with reduced suitability for spittlebug nymphal development might indeed limit local recruitment while preserving soil protection and ecosystem services. To our knowledge, Morente et al. (2022) were the first to propose this strategy in the European context, demonstrating differential host suitability under controlled conditions. However, field-based evidence across diverse vegetational assemblages remains limited (Bodino et al. 2019, Dongiovanni et al. 2019), constraining the development of practical recommendations for farmers. Improved knowledge of vector juveniles host plant use under field conditions is therefore essential for designing vegetation-based vector management strategies compatible with IPM principles. Strategic selection of less suitable ground cover species could reduce nymphal survival and subsequent adult pressure while maintaining soil stability, biodiversity, and long-term agroecosystem sustainability. In this study, we report on host plant use by juveniles of *P. spumarius* and *N. campestris* based on a large-scale survey conducted across 105 one-hectare olive orchard plots in Apulia, southeastern Italy. Our findings provide applied evidence to support the development of selective vegetation management strategies as alternatives to conventional ground cover removal practices.

## Materials and Methods

### Description of the Sampling Sites

**Fig. 1.**
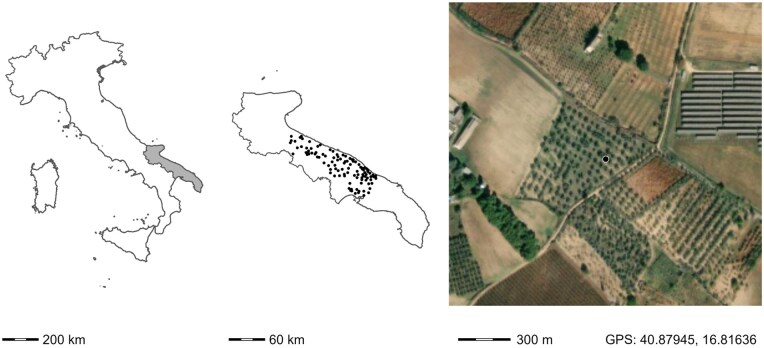
Map of the sampling sites, Apulia Region (South-East Italy, 40°47′35″ N, 17°06′03″ E).

The survey was conducted on 105 olive groves in the Apulia Region (South-East Italy, 40°47′34.51″ N, 17°06′03.44″ E) of approximately 1 ha each (Fig. 1), selected in order to ensure variability of landscape, altitude, spatial, micro-climatic conditions and maintenance practices, reflecting differences in the spontaneous plant species. For olive groves larger than 1 ha, the survey was conducted on a randomly selected 1 ha portion. Because sites could not be sampled at uniform plant phenological stages, surveys were conducted under slightly different phenological conditions across sites.

### Sampling of *P. spumarius* Nymphs

Data on juvenile frequency and abundance on herbaceous species composing the olive groves ground cover were collected through conservative (non-destructive) sampling for approximately 2 months (20 February to 22 May 2024). Each site/olive grove was monitored every 10 days, with 14 total sampling rounds per year per site. In each site, we examined spittlebug juveniles and herbaceous plant populations on five 0.25 m^2^ transects (Supplementary Fig. S1), randomly positioned across the olive groves, considering only transects where at least a spittle mass was detected. For each transect, we recorded: (i) the total number of spittle masses per plant; (ii) the total number of nymphs per spittle per plant, sorted by instar.

The “average nymph abundance” per host plant species was then calculated as the total number of nymphs (independently of the instar) recorded on all the specimens of a given plant species recorded during the campaign, divided by the total number of times that the plant species was observed inside the transects throughout the sampling campaign. The “occurrence rate” was expressed as the number of times that a species was observed hosting at least a nymph (independently of the number of nymphs/spittles per plant) divided by the total number of times that the plant species was observed inside the transects throughout the sampling campaign.

The sampling protocol was conservative: after visual detection, the nymphs were gently removed with a wet paintbrush, counted, sorted by instars, and released. All the plant species recorded in the transects, whether hosting or not spittlebug juveniles, were collected and identified in the lab using the digital taxonomic keys for plants identification provided by Pignatti (2019). In case of uncertain identification due to a lack of taxonomic characteristics in the early stages of plant growth, plants were categorized at least to the genus or family level. To verify species identity, 5% of the collected nymphs were individually caged on periwinkle (*Catharanthus roseus*) and *Bromus spp*. plants (for *P. spumarius* and *N. campestris*, respectively) within insect rearing cages (Omnes Artes GA20, 35 × 35 × 60 cm), maintained under field conditions in the olive orchard where the nymphs were collected. Nymphs were left to develop until adult emergence. Upon emergence, adults were collected, preserved in 70% ethanol, and transported to the laboratory for species identification using the morphological taxonomic keys provided by Ossiannilsson (1981).

### Statistical Analysis

To evaluate host plant use exhibited by *P. spumarius* and *N. campestris* nymphs, generalized linear mixed models (GLMMs) with a negative binomial distribution (ln link function) were fitted using R software (R Core Team 2025). The response variables were the total abundance of *P. spumarius* nymphs (all instars combined) and the abundance of each individual nymphal instar (1st and 2nd instars were pooled and analyzed together to avoid convergence issues due to excess zeros) per sampling unit, ie the plant species within each transect. In all models, host plant species was included as a categorical fixed effect, while site and transect nested within site were included as random effects. Each transect was sampled on a single date. Host plant species on which no nymphs were recorded were excluded from the analyses. Additionally, host plant species represented by fewer than 15 observations were removed to ensure statistical robustness (Legendre and Legendre 1998). This threshold was selected because models fitted with lower thresholds showed convergence difficulties. After filtering the 194 plant species present in the complete dataset, 71 species observed in 15 or more transects retained for data analysis, while 123 less represented species were excluded. Pairwise comparisons among host plant species were not performed due to the high number of levels in the explanatory variable, which would have resulted in an excessive number of comparisons and an increased risk of type I error. Identical analyses were conducted for *N. campestris* nymphs (all instars combined due to low instar-specific abundance). All the analyses were carried out in R (R Core Team 2025). Models were fitted using the “glmmTMB” package (Brooks et al. 2017). Estimates and standard errors of the abundance of nymphs for each plant species obtained using the “emmeans” package (Lenth 2025). Models were checked for overdispersion and residual distribution using the “DHARMa” package (Hartig 2024). Barplots were created using the package “ggplot2” (Wickham 2016).

## Results

For each insect species, the number of sites (Ns), transects (Nt), and observations (No) included in the analyses is reported (Table 1).

**Table 1.** Output of generalized linear mixed models

*Philaenus spumarius* (Ns = 90, Nt = 687, No = 4486)			
	χ^2^	df	*P* value
**All instars**	546.02	66	<.001
**I-II instar**	200.86	66	<.001
**III instar**	116.96	66	<.001
**IV instar**	147.66	66	<.001
**V instar**	128.89	66	<.001
***Neophilaenus campestris* (Ns = 60, Nt = 278, No = 2104)**			
**All instars**	101.44	13	<.001

Analysis of deviance from GLMMs testing the effect of host plant species on *Philaenus spumarius* and *Neophilaenus campestris* nymphs, considering all instars combined and by instar (I and II instars were pooled and analyzed together). For *N. campestris*, analyses were performed only on combined instars due to low abundance. For each insect species, the number of sites (Ns), transects (Nt), and observations (No) included in the analyses is reported. Type II Wald chi-square tests (χ^2^), degrees of freedom (df), and *P* value are provided for all models.

### Philaenus spumarius

*P. spumarius* nymphs were observed on a total of 131 plant species; a total of 2,510 *P. spumarius* nymphs were identified and counted throughout the survey. On the other hand, meadow spittlebug nymphs were never observed on 84 of the plant species present in the 105 olive orchards surveyed (Supplementary Table S1).

Overall, we calculated juvenile abundance and occurrence on the 71 most frequently occurring species in Apulian olive groves, ie plant species observed at least 15× during the sampling campaign (Supplementary Table S1). Considering all the plant species specimens observed within the transects (4,652), a total of 2,081 nymphs were counted, with an occurrence rate of 44.73% (frequency of spittlebugs presence on all the host species). The full list reporting abundance and occurrence of nymphs per instar per plant species is reported in Supplementary Material (Supplementary Table S1).

Plants belonging to the family Asteraceae were the most occurring favorite hosts, displaying an occurrence rate of 36.3%, and an average nymph abundance of 0.68 (1,154 total nymphs counted), followed by Fabaceae (29.6% occurrence rate, average nymph abundance of 0.41, 466 nymphs in total), Poaceae (29% occurrence rate, average nymph abundance of 0.33, 285 nymphs), and Apiaceae (27.6% occurrence rate, average nymph abundance of 0.53, 184 nymphs). The full tabulation of nymphs per instar per plant families is reported in Supplementary Material (Supplementary Table S1).

Considering the hosts at species level, *Sonchus asper* resulted the preferred plant (71.7% occurrence rate, average nymph abundance of 1.97, 118 nymphs), followed by *Foeniculum vulgare* (72% occurrence rate, average nymph abundance of 1.80, 45 nymphs), *Urospermum dalechampii* (81.3% occurrence rate, average nymph abundance of 1.56, 25 nymphs), *Erigeron canadensis* (73.3% occurrence rate, average nymph abundance of 1.32, 99 nymphs), and *Calendula arvensis* (56.4% occurrence rate, average nymph abundance of 1.07, 386 nymphs) (Table 2).

**Table 2.** *Philaenus spumarius* nymphs—plant species suitability

Host plant species	Plant occurrence	Nymph abundance	Mean ± SE	Occurrence rate (%)	1–2 instar (%)	3rd instar (%)	4th instar (%)	5th instar (%)
***Foeniculum vulgare* (Apiaceae)**	25	45	1.41 ± 0.40	72	42.2	28.9	26.7	2.2
***Sonchus asper* (Asteraceae)**	60	118	1.26 ± 0.22	71.7	24.6	27.1	33.9	14.4
***Urospermum dalechampii* (Asteraceae)**	16	25	1.15 ± 0.40	81.3	20	12	12	56
***Erigeron canadensis* (Asteraceae)**	75	99	1.09 ± 0.23	73.3	53.5	22.2	4	20.2
***Calendula arvensis* (Asteraceae)**	360	386	0.5 ± 0.06	56.4	46.9	34.5	13.7	4.9
***Euphorbia helioscopia* (Euphorbiaceae)**	37	1	0.02 ± 0.02	2.7	0	100	0	0
***Bromus diandrus* (Poaceae)**	41	1	0.02 ± 0.02	2.4	0	0	0	100
***Lysimachia foemina* (Primulaceae)**	58	1	0.01 ± 0.01	3.4	0	100	0	0
***Papaver rhoeas* (Papaveraceae)**	40	1	0.02 ± 0.02	2.5	0	100	0	0
***Senecio vulgaris* (Asteraceae)**	57	1	0.01 ± 0.01	1.8	100	0	0	0
*Astragalus hamosus* (Fabaceae)*	66	0	0.00 ± 0.00	0	NA	NA	NA	NA
*Fumaria parviflora* (Papaveraceae)*	16	0	0.00 ± 0.00	0	NA	NA	NA	NA
*Mercurialis annua* (Euphorbiaceae)*	26	0	0.00 ± 0.00	0	NA	NA	NA	NA
*Muscari spp.* (Liliaceae)*	52	0	0.00 ± 0.00	0	NA	NA	NA	NA
*Oxalis pes-caprae* (Oxalidaceae)*	21	0	0.00 ± 0.00	0	NA	NA	NA	NA

Top 5 and least suitable host plant species of *P. spumarius* nymphs. Mean estimates ± standard errors derived from generalized linear mixed models are shown. Asterisks indicate species identified at family level and non-host plants not included in statistical analysis.

Abbreviation: NA = not applicable.

**Fig. 2.**
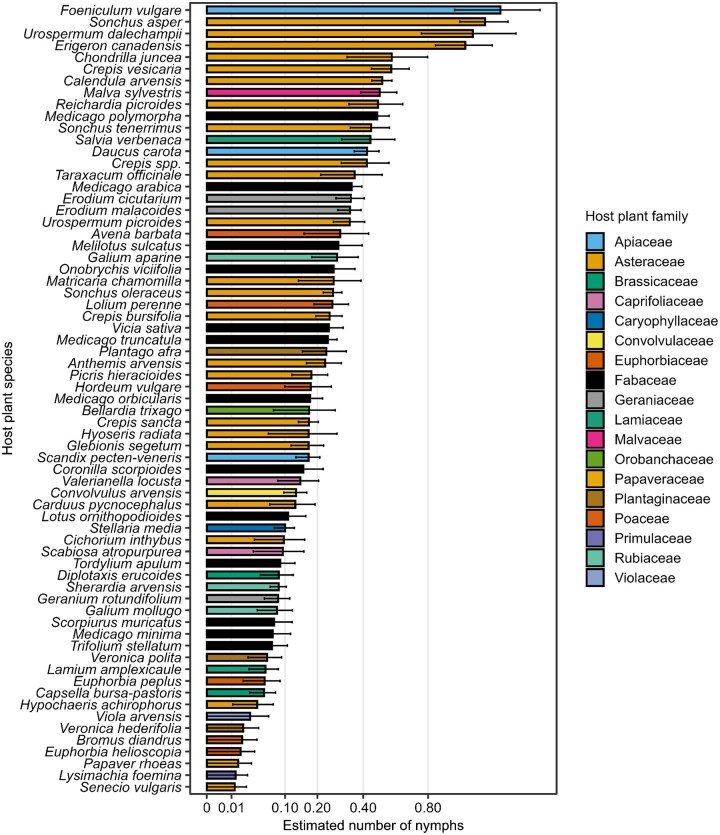
Effect of host plant species on *Philaenus spumarius* nymph abundance. Mean estimates ± standard errors derived from generalized linear mixed models are shown.

Throughout the campaign, considering only the plant species observed more than 15×, the lowest-response species in nymph suitability included *Senecio vulgaris*, *Lysimachia foemina*, *Papaver rhoeas*, and *Euphorbia helioscopia*, all of which showed estimated nymph abundance and occurrence rates below 0.04. These values were significantly lower compared with high-response species, such as *F. vulgare*, *U. dalechampii*, *S. asper*, and *E. canadensis*, which showed the highest estimated nymph abundance (Tables 1 and 2, Fig. 2). Notably, despite being relatively well represented in the dataset (ie >15 occurrences), *P. spumarius* nymphs were never detected on *Oxalis pes-caprae* (21 specimens), *Astragalus hamosus* (66), *Fumaria parviflora* (16), *Muscari spp*. (52), or *Mercurialis annua* (26).

Instar-specific models revealed significant differences in estimated nymph abundance among host plants across all 5 instars (Supplementary Figs S2–S5; Table 1; Supplementary Table S3). For some plant species, nymph abundance was higher in early instars (1st–2nd), whereas in others it increased in later developmental stages. This pattern is confirmed by raw data on the proportional composition of instars for each host species (see Table 2; Supplementary Tables S1 and S4), indicating a shift in host association along nymphal development. For instance, *Foeniculum vulgare* ranked among the top three host species from 1st-2nd to 4th instar but dropped markedly in 5th instar (nymph abundance and instars proportion in percentages, respectively: 1st-2nd: 19, 42.2%; 3rd: 13, 28.9%; 4th: 12, 26.7%; 5th: 1, 2.2%). A similar trend was observed for *Calendula arvensis* (1st-2nd: 181, 46.9%; 3rd: 133, 34.5%; 4th: 53, 13.7%; 5th: 19, 4.9%) and *Crepis sancta* (1st-2nd: 40, 95%; 3rd: 1, 2.4%; 4th: 1, 2.4%; 5th: 0, 0%), which predominantly hosted early instars. In contrast, for species such as *Cichorium intybus*, only later instars were recorded (nymph abundance and instar proportions: 1st-2nd: 0, 0%; 3rd: 0, 0%; 4th: 5, 38.5%; 5th: 8, 61.5%). This pattern reflects its later emergence during the survey period due to delayed flowering, which resulted in the absence of 1st and 2nd instar nymphs, as these early developmental stages occurred prior to plant availability.

### Neophilaenus campestris

**Fig. 3.**
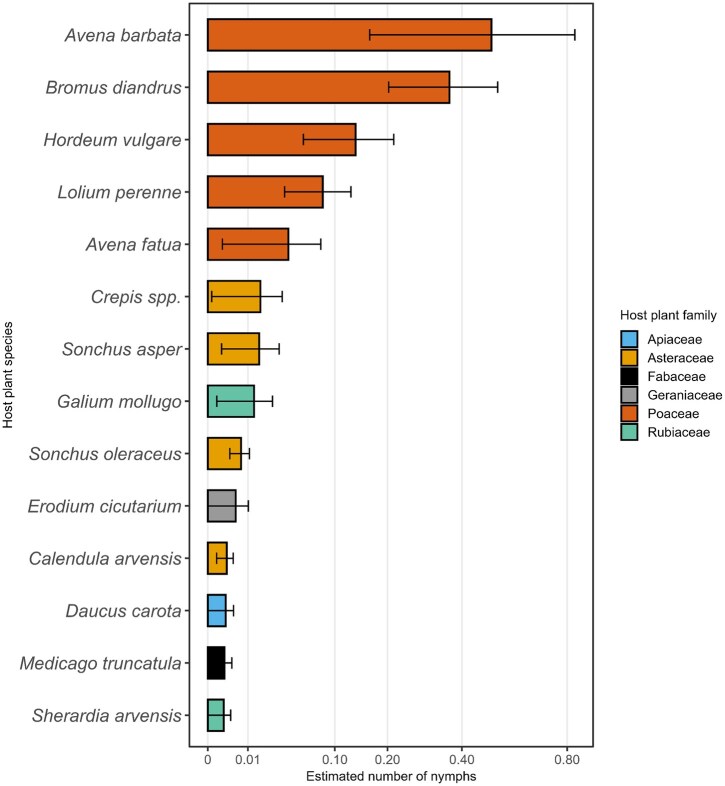
Effect of host plant species on *Neophilaenus campestris* nymph abundance. Mean estimates ± standard errors derived from generalized linear mixed models are shown.

For *N. campestris*, 1,150 of the total 1,176 nymphs recorded during the entire survey (97.7%) were found on plants belonging to the Poaceae family, and specifically on 14 plant species (Table 3, Supplementary Table S2). However, we excluded from the statistical analysis the Poaceae species identified only at family level due to the lack of easily recognizable taxonomic features in their first phenological stages (corresponding to spittlebug juvenile occurrence on these plants under field conditions). None of the other 62 species observed at least 15× during the sampling campaign (same criterion applied to the meadow spittlebug, see details above) hosted *N. campestris* nymphs (Supplementary Table S2). General linear mixed models showed significant differences among host plant species of *N. campestris* in their host suitability (Table 1), estimated considering nymph abundance on each species. The least suitable species for *N. campestris* juveniles were *Sherardia arvensis*, *Medicago truncatula*, *Daucus carota*, and *Calendula arvensis*, all showing values of estimated nymph abundance below 0.01 and occurrence rate ≃0 (Table 3; Fig. 3). These values were significantly lower compared to high-response (most suitable) species, such as *Avena barbata* (0.5 estimated nymph abundance), *Bromus diandrus* (0.36), and *Hordeum vulgare* (0.14) (Table 1).

**Table 3.** *Neophilaenus campestris* nymphs—plant species suitability

Host plant species	Plant occurrence	Nymph abundance	Mean ± SE	Occurrence rate (%)	1–2 instar (%)	3rd instar (%)	4th instar (%)	5th instar (%)
Poaceae*	877	1,046	NA	94.5	45.4	25.1	21.9	7.6
***Avena barbata* (Poaceae)**	24	25	0.50 ± 0.34	66.7	0	8	60	32
***Bromus diandrus* (Poaceae)**	64	33	0.36 ± 0.16	46.9	15.2	3	33.3	48.5
***Hordeum vulgare* (Poaceae)**	58	18	0.14 ± 0.08	13.8	38.9	33.3	22.2	5.6
***Lolium perenne* (Poaceae)**	58	9	0.08 ± 0.05	15.5	11	0	44.5	44.5
***Avena fatua* (Poaceae)**	30	2	0.04 ± 0.04	6.7	0	0	100	0
***Erodium cicutarium* (Geraniaceae)**	108	1	0.00 ± 0.01	≈0	100	0	0	0
***Calendula arvensis* (Asteraceae)**	456	2	0.00 ± 0.00	≈0	100	0	0	100
***Daucus carota* (Apiaceae)**	235	1	0.00 ± 0.00	≈0	0	0	0	100
***Medicago truncatula* (Fabaceae)**	255	1	0.00 ± 0.00	≈0	0	100	0	0
***Sherardia arvensis* (Rubiaceae)**	299	1	0.00 ± 0.00	≈0	0	100	0	0

Top 5 and least suitable host plant species of *Neophilaenus campestris* nymphs, grouped by family. Mean estimates ± standard errors derived from generalized linear mixed models are shown. Asterisks indicate species identified at family level (pooled records of specimens not necessarily belonging to the same botanical species) and not included in statistical analysis.

Abbreviation: NA = not applicable.

## Discussion

This study provides large-scale field evidence that herbaceous plant species differ markedly in their suitability for nymphal development of *P. spumarius* and *N. campestris*, two competent vector species of *X. fastidiosa* widely involved in European outbreaks. Across 105 olive orchards, we showed that ground cover composition rather than mere ground cover presence or incidence is a key ecological driver of spittlebugs recruitment. These findings provide empirical support for shifting from generalized ground cover removal toward selective vegetation management based on plant suitability for juvenile development. Mechanical removal of herbaceous vegetation and repeated tillage are currently implemented to suppress nymphal populations during peak development. However, such practices can negatively affect soil structure, reduce organic matter, increase erosion risk, and decrease arthropod biodiversity, particularly in Mediterranean agroecosystems already vulnerable to drought and land degradation (Pimentel and Burgess 2013, García-Ruiz et al. 2015, Sánchez-Bayo and Wyckhuys 2019, Avosani et al. 2023). Vegetation disturbance can also disrupt natural enemy communities and associated ecosystem services (Landis et al. 2000, Bianchi et al. 2006), potentially compromising long-term pest regulation. Furthermore, complete removal of ground vegetation may not sustainably suppress local populations and could even enhance immigration of spittlebug adults from surrounding habitats. Increased adult recruitment into managed fields may elevate probing activity and, in areas affected by *X. fastidiosa*, increase the probability of pathogen transmission events (Davis et al. 2009, Lazar et al. 2025).

Consistent with previous field studies (Bodino et al. 2019, Dongiovanni et al. 2019), juveniles of both spittlebug species were heterogeneously distributed across plant taxa. However, our results indicate that host suitability was primarily species-specific rather than determined at the family level. Even within the most frequently colonized family (Asteraceae), substantial variability was observed, with some species consistently avoided by juveniles. This pattern suggests that plant functional traits operating at the species level, rather than broad taxonomic affiliation, drive host acceptance and nymphal performance. Although *P. spumarius* is highly polyphagous (Thompson et al. 2023), nymphs were recorded only on a subset of available plant species, and 84 species present in surveyed orchards never hosted juveniles. Restricting analysis to plant species observed at least 15× during the monitoring campaign to avoid bias from occasional records, meadow spittlebug nymphs were never observed on *O. pes-caprae*, *A. hamosus*, *M. annua*, *F. parviflora*, and *Muscari spp*., and occurred at very low abundance on *S. vulgaris*, *L. foemina*, *P. rhoeas*, and *E. helioscopia*. Likewise, *N. campestris* exhibited a striking ecological pattern, with 97.7% of juveniles developing on Poaceae and almost complete absence from dicotyledonous species. Such marked differences in juvenile abundance among plant *taxa* likely reflect variation in functional traits influencing feeding success, nutritional suitability, and microhabitat conditions, mechanisms not directly tested in the present study. Spittlebug nymphs are obligate xylem sap feeders, and xylem chemistry represents a fundamental determinant of host acceptance and performance (Wiegert 1964, Sisterson et al. 2017, Cornara et al. 2024, Tinelli et al. 2025). Although xylem sap generally contains lower concentrations of secondary metabolites than phloem, it remains nutritionally dilute and chemically heterogeneous among plant species (Wiegert 1964, Tzin and Galili 2010). Differences in amino acid composition, inorganic ion balance, and phenolic content can influence ingestion rates and insect performance in xylem-feeding Hemipterans (Backus 1985, Andersen et al. 1989). For instance, species within Euphorbiaceae (eg *M. annua* and *E. helioscopia*) and Fabaceae (eg *A. hamosus*) produce alkaloids, terpenoids, and phenolic compounds that may interfere with herbivore feeding or physiology (Després et al. 2007). Even broadly polyphagous insects may avoid nutritionally suboptimal or chemically defended hosts when more suitable alternatives are available (Ali and Agrawal 2012). Moreover, these physiological constraints may help explain the marked ecological specialization observed in *N. campestris*, whose strong association with Poaceae suggests adaptation to monocot-specific xylem properties. Grasses differ substantially from dicots in vascular organization and hydraulic architecture. Monocot xylem exhibits distinct vessel distribution and hydraulic properties compared with dicotyledonous plants (Sperry et al. 2006), and nitrogen allocation patterns during early vegetative growth can differ markedly between grasses and dicots (Tzin and Galili 2010). Adaptation to monocot-specific xylem chemistry may therefore limit the ability of *N. campestris* to efficiently exploit dicot hosts, even when these are abundant within the same habitat.

Plant architectural traits could be an additional, or pivotal component underpinning host plant use. For *P. spumarius*, plants lacking basal shelter, dense leaf arrangement, or ground-level structures may expose nymphs to increased thermal and desiccation stress. Although spittle masses mitigate water loss, microclimatic exposure remains a critical factor affecting juvenile survival, particularly under Mediterranean spring conditions characterized by rapid warming and intermittent drought (Jansa et al. 2017). In contrast, basal rosettes and hemicryptophytic growth forms, common within Asteraceae, provide humid and structurally protected microhabitats near the soil surface, likely favoring nymph persistence (Bodino et al. 2019). Similarly, the strong association of *N. campestris* with grasses suggests adaptation to the linear and tillering architecture of Poaceae. Dense basal sheaths and narrow vertical leaves create shaded and relatively humid microenvironments at ground level, facilitating stable attachment of spittle masses and reducing direct exposure to solar radiation. Moreover, the relatively uniform vertical structure of grass stands may lower probing costs and improve feeding efficiency for a species adapted to monocot hosts. Comparable links between plant architecture and host specialization have been reported in other spittlebug species (McEvoy 1986). However, this study did not directly assess the extent to which these traits influence host plant use by spittlebug nymphs. Further physiological and behavioral investigations will be necessary to clarify their relative contribution to host use patterns.

Temporal dynamics in plant phenology likely represent an additional key driver of host plant use patterns. Plant functional traits are not static over the course of spittlebug nymphal development and shifts in phenological stages can alter both plant chemistry and physiological properties (Agerbirk et al. 2001), including nutrient availability, water content, and the concentration of secondary metabolites relevant to xylem-feeding insects. At the same time, seasonal turnover in plant communities modifies species availability and relative abundance within agroecosystems, progressively reshaping the set of potential hosts. These dynamics are likely to affect differentially early and late nymphal instars, explaining the shifts in host association observed in our results. For example, plant species that emerge or reach suitable developmental stages later in the season may only support advanced instars, whereas early-emerging species are more likely to host younger nymphs. In addition, because sampling sites were not phenologically synchronized, plant species recorded within the same sampling rounds were often at different developmental stages across sites. As a result, nymphs likely encountered hosts that differed not only in species identity but also in physiological condition and structural suitability. Additionally, climatic and microclimatic differences among sampling sites may have further contributed to the observed variability in host plant use by shaping local plant community composition. Environmental variables such as temperature, precipitation, soil moisture, solar exposure, and local humidity can act as ecological filters influencing the diversity and composition of arable plant communities (Fanfarillo et al. 2023). Consistent with this, the differences in plant assemblages observed among our study sites may partially reflect local climatic and microclimatic conditions, which likely affected the availability and relative abundance of potential host plants throughout the sampling period. Consequently, part of the variation observed in nymph abundance and host associations among sites may result from environmentally mediated differences in plant community structure and host suitability, rather than from intrinsic plant species traits alone. Finally, juvenile abundance and occurrence might also reflect oviposition patterns rather than solely host plant suitability, particularly during early developmental stages. After egg hatching, 1st instar nymphs must locate and settle rapidly on a suitable host (Weaver and King 1954), allowing nymphs to find a shelter and reduce exposure to solar radiation and desiccation. Therefore, the occurrence of first instar individuals is likely influenced by the spatial distribution of oviposition sites and the proximity of suitable plants to preferred egg-laying substrates, rather than by active host selection. Under these constraints, early instar distribution is expected to reflect the oviposition patterns and environmental conditions affecting overwintering eggs, rather than independent host choice (Whittaker 1973).

From an epidemiological perspective, the epidemiology of *X. fastidiosa* in Mediterranean agroecosystems including olive orchards is strongly influenced by vector abundance and dispersal patterns (Bodino et al. 2023). Juvenile suppression at the ground cover stage represents a strategic intervention point, as nymphs are relatively immobile and spatially predictable. Our findings indicate that selective vegetation management, minimizing grass-dominated cover (thus reducing the prevalence of *N. campestris* preferred hosts), and promoting dicot species exhibiting low suitability for the meadow spittlebug development such as *O. pes-caprae*, *A. hamosus*, *M. annua*, *F. parviflora*, *Muscari* spp., *S. vulgaris*, *L. foemina*, *P. rhoeas*, and *E. helioscopia*, may reduce local population buildup while preserving soil protection and ecological functions. Such strategies align with agroecological “push–pull” principles (Cook et al. 2007), where habitat manipulation modifies pest behavior and survival rather than relying solely on disturbance-based suppression. Importantly, vegetation-based management does not aim to eradicate spittlebugs, which are native components of Mediterranean ecosystems, but to reduce vector pressure within agroecosystems by altering habitat suitability. This approach represents a shift from reactive vector control toward preventive agroecosystem design.

Although our survey provides robust field-scale evidence of differential host suitability, causal mechanisms require experimental validation. Controlled assays comparing nymph survival, development, and adult emergence across plant species, as well as studies linking juvenile suppression to adult density and *X. fastidiosa* transmission rates, are essential to quantify the epidemiological impact of vegetation-based control strategies. Equally important will be evaluating effects on non-target organisms to ensure that proposed management practices enhance, rather than compromise, overall agroecosystem resilience.

## Supplementary Material

toag179_Supplementary_Data

## Data Availability

Additional data are available from the corresponding author (giada.spadavecchia@uniba.it) upon request.
