# Supplementary material for: Host plant use by spittlebug nymphs in agroecosystems: a field survey supporting targeted vegetation management
Source: J Econ Entomol. 2026 Jun 27;119(4):2422–31. doi: 10.1093/jee/toag179 (PMC13462268; doi:10.1093/jee/toag179)
Supplement: Supplementary file 1 [file toag179_supplementary_data.docx]

**Host plant use by spittlebug nymphs in agroecosystems: a field survey supporting targeted vegetation management**

Maurizio Vincenzo Puzzovivo^1,2^, Giacomo Santoiemma^3^, Giada Spadavecchia^1*^, Nicola Tagarelli^2^, Maria Luisa Vitale^2^, Rosalia Marroccoli^1^, Vincenzo Verrastro^2^, Vinton Thompson^5^, Daniele Cornara^1^

1. Department of Soil, Plant and Food Science (DiSSPA), University of Bari Aldo Moro, Via Amendola 165/A, 70126 Bari, Italy
2 International Centre for Advanced Mediterranean Agronomic Studies – Institute of Bari (CIHEAM Bari), Via Ceglie 9, 70010 Valenzano (Bari), Italy
3 DAFNAE Department, University of Padua, Viale dell’Università 16, 35020 Legnaro, Italy
4 French National Institute for Agricultural Research (INRA), UMR1355-ISA, 400 route des chappes, 06903 Sophia-Antipolis, France
5 American Museum of Natural History, 200 Central Park West, New York, NY 10024, United States

*Corresponding author: giada.spadavecchia@uniba.it

**Figures**

**Figure S1.** Transect sampling of spittlebugs nymphs.

**Figure S2**. Effect of host plant species on *Philaenus spumarius* 1^st^-2^nd^ instar nymph abundance. Mean estimates ± standard errors derived from generalized linear mixed models are shown.

 **Figure S3.** Effect of host plant species on Philaenus spumarius 3^rd^ instar nymph abundance. Mean estimates ± standard errors derived from generalized linear mixed models are shown.

**Figure S4.** Effect of host plant species on Philaenus spumarius 4^th^ instar nymph abundance. Mean estimates ± standard errors derived from generalized linear mixed models are shown.

**Figure S5.** Effect of host plant species on Philaenus spumarius 5^th^ instar nymph abundance. Mean estimates ± standard errors derived from generalized linear mixed models are shown.

**Supplementary tables**

**Table S1. Philaenus spumarius nymphs - plant species suitability.** List of host plant species (ranked by estimated nymph abundance) and non-suitable species of P. spumarius nymphs. Asterisks indicate species not included in statistical analysis.

| **Transect species** | **Plant occurrences in transects** | **Occurrences of *P. spumarius*** | **Nymph abundance** | **Mean ± SE** | **Occurrence rate %** | **1st–2nd instar %** | **3rd instar %** | **4th instar %** | **5th instar %** |
| --- | --- | --- | --- | --- | --- | --- | --- | --- | --- |
| *Foeniculum vulgare* | 25 | 18 | 45 | 1.41 ± 0.41 | 72,0 | 42.2 | 28.9 | 26.7 | 2.2 |
| *Sonchus asper* | 60 | 43 | 118 | 1.26 ± 0.22 | 71,7 | 24.6 | 27.1 | 33.9 | 14.4 |
| *Urospermum dalechampii* | 16 | 13 | 25 | 1.16 ± 0.4 | 81,3 | 20 | 12 | 12 | 56 |
| *Erigeron canadensis* | 75 | 55 | 99 | 1.09 ± 0.24 | 73,3 | 53.5 | 22.2 | 4 | 20.2 |
| *Cichorium inthybus* | 16 | 8 | 13 | 0.56 ± 0.24 | 50,0 | 0 | 0 | 38.5 | 61.5 |
| *Crepis vesicaria* | 69 | 31 | 58 | 0.56 ± 0.11 | 44,9 | 75.9 | 12.1 | 6.9 | 5.2 |
| *Calendula arvensis* | 360 | 203 | 386 | 0.5 ± 0.06 | 56,4 | 46.9 | 34.5 | 13.7 | 4.9 |
| *Malva sylvestris* | 61 | 23 | 58 | 0.49 ± 0.1 | 37,7 | 29.3 | 17.2 | 10.3 | 43.1 |
| *Reichardia picroides* | 25 | 8 | 21 | 0.48 ± 0.15 | 32,0 | 61.9 | 28.6 | 4.8 | 4.8 |
| *Medicago polymorpha* | 181 | 92 | 140 | 0.47 ± 0.07 | 50,8 | 20.7 | 18.6 | 14.3 | 46.4 |
| *Salvia verbenaca* | 29 | 7 | 17 | 0.44 ± 0.14 | 24,1 | 35.3 | 0 | 11.8 | 52.9 |
| *Sonchus tenerrimus* | 53 | 26 | 43 | 0.44 ± 0.1 | 49,1 | 55.8 | 9.3 | 7 | 27.9 |
| *Crepis spp.* | 32 | 11 | 22 | 0.42 ± 0.12 | 34,4 | 77.3 | 22.7 | 0 | 0 |
| *Daucus carota* | 167 | 52 | 93 | 0.42 ± 0.06 | 31,1 | 30.1 | 28 | 19.4 | 22.6 |
| *Taraxacum officinale* | 16 | 6 | 10 | 0.36 ± 0.15 | 37,5 | 0 | 60 | 30 | 10 |
| *Erodium cicutarium* | 91 | 27 | 54 | 0.34 ± 0.07 | 29,7 | 48.1 | 24.1 | 20.4 | 7.4 |
| *Medicago arabica* | 194 | 90 | 132 | 0.34 ± 0.05 | 46,4 | 59.1 | 30.3 | 8.3 | 2.3 |
| *Erodium malacoides* | 150 | 50 | 84 | 0.34 ± 0.05 | 33,3 | 32.1 | 26.2 | 16.7 | 25 |
| *Urospermum picroides* | 81 | 21 | 35 | 0.33 ± 0.07 | 25,9 | 11.4 | 48.6 | 28.6 | 11.4 |
| *Avena barbata* | 22 | 6 | 6 | 0.29 ± 0.14 | 27,3 | 0 | 0 | 16.7 | 83.3 |
| *Melilotus sulcatus* | 20 | 7 | 11 | 0.28 ± 0.11 | 35,0 | 45.5 | 9.1 | 18.2 | 27.3 |
| *Galium aparine* | 33 | 11 | 12 | 0.28 ± 0.1 | 33,3 | 41.7 | 25 | 25 | 8.3 |
| *Matricaria chamomilla* | 20 | 4 | 6 | 0.26 ± 0.13 | 20,0 | 33.3 | 50 | 0 | 16.7 |
| *Onobrychis viciifolia* | 29 | 11 | 11 | 0.26 ± 0.1 | 37,9 | 9.1 | 18.2 | 36.4 | 36.4 |
| *Lolium perenne* | 54 | 23 | 23 | 0.26 ± 0.07 | 42,6 | 21.7 | 13 | 39.1 | 26.1 |
| *Sonchus oleraceus* | 237 | 62 | 90 | 0.26 ± 0.04 | 26,2 | 17.8 | 18.9 | 17.8 | 45.6 |
| *Crepis bursifolia* | 86 | 19 | 45 | 0.25 ± 0.05 | 22,1 | 86.7 | 0 | 4.4 | 8.9 |
| *Vicia sativa* | 76 | 20 | 24 | 0.24 ± 0.06 | 26,3 | 29.2 | 25 | 20.8 | 25 |
| *Medicago truncatula* | 217 | 58 | 75 | 0.24 ± 0.04 | 26,7 | 26.7 | 26.7 | 18.7 | 28 |
| *Plantago afra* | 39 | 12 | 11 | 0.23 ± 0.08 | 30,8 | 9.1 | 9.1 | 36.4 | 45.5 |
| *Anthemis arvensis* | 41 | 11 | 22 | 0.23 ± 0.07 | 26,8 | 4.5 | 18.2 | 31.8 | 45.5 |
| *Hordeum vulgare* | 23 | 6 | 7 | 0.18 ± 0.08 | 26,1 | 0 | 28.6 | 42.9 | 28.6 |
| *Picris hieracioides* | 49 | 6 | 12 | 0.18 ± 0.06 | 12,2 | 16.7 | 50 | 8.3 | 25 |
| *Hyoseris radiata* | 18 | 2 | 3 | 0.17 ± 0.11 | 11,1 | 33.3 | 33.3 | 33.3 | 0 |
| *Bellardia trixago* | 15 | 3 | 4 | 0.17 ± 0.1 | 20,0 | 0 | 25 | 75 | 0 |
| *Glebionis segetum* | 51 | 11 | 14 | 0.17 ± 0.05 | 21,6 | 35.7 | 57.1 | 7.1 | 0 |
| *Scandix pecten-veneris* | 93 | 15 | 28 | 0.17 ± 0.04 | 16,1 | 82.1 | 10.7 | 0 | 7.1 |
| *Medicago orbicularis* | 74 | 16 | 23 | 0.17 ± 0.04 | 21,6 | 0 | 34.8 | 17.4 | 47.8 |
| *Crepis sancta* | 159 | 28 | 42 | 0.17 ± 0.03 | 17,6 | 95.2 | 2.4 | 2.4 | 0 |
| *Coronilla scorpioides* | 31 | 6 | 6 | 0.15 ± 0.07 | 19,4 | 33.3 | 33.3 | 0 | 33.3 |
| *Valerianella locusta* | 29 | 5 | 7 | 0.14 ± 0.06 | 17,2 | 71.4 | 0 | 0 | 28.6 |
| *Carduus pycnocephalus* | 27 | 2 | 5 | 0.13 ± 0.06 | 7,4 | 0 | 0 | 0 | 100 |
| *Convolvulus arvensis* | 93 | 16 | 22 | 0.13 ± 0.03 | 17,2 | 68.2 | 27.3 | 0 | 4.5 |
| *Lotus ornithopodioides* | 40 | 5 | 5 | 0.11 ± 0.05 | 12,5 | 20 | 40 | 20 | 20 |
| *Chondrilla juncea* | 22 | 3 | 3 | 0.1 ± 0.06 | 13,6 | 100 | 0 | 0 | 0 |
| *Stellaria media* | 121 | 17 | 21 | 0.1 ± 0.03 | 14,0 | 85.7 | 0 | 0 | 14.3 |
| *Scabiosa atropurpurea* | 22 | 3 | 3 | 0.09 ± 0.06 | 13,6 | 33.3 | 33.3 | 0 | 33.3 |
| *Tordylium apulum* | 51 | 6 | 6 | 0.09 ± 0.04 | 11,8 | 83.3 | 0 | 16.7 | 0 |
| *Diplotaxis erucoides* | 48 | 4 | 6 | 0.08 ± 0.04 | 8,3 | 83.3 | 0 | 0 | 16.7 |
| *Galium mollugo* | 39 | 5 | 5 | 0.08 ± 0.04 | 12,8 | 80 | 20 | 0 | 0 |
| *Geranium rotundifolium* | 84 | 6 | 10 | 0.08 ± 0.03 | 7,1 | 90 | 0 | 10 | 0 |
| *Sherardia arvensis* | 212 | 20 | 27 | 0.08 ± 0.02 | 9,4 | 63 | 33.3 | 3.7 | 0 |
| *Scorpiurus muricatus* | 26 | 4 | 3 | 0.07 ± 0.05 | 15,4 | 0 | 33.3 | 0 | 66.7 |
| *Trifolium stellatum* | 55 | 3 | 4 | 0.07 ± 0.04 | 5,5 | 25 | 25 | 25 | 25 |
| *Medicago minima* | 29 | 3 | 3 | 0.07 ± 0.04 | 10,3 | 0 | 33.3 | 33.3 | 33.3 |
| *Lamium amplexicaule* | 47 | 3 | 5 | 0.06 ± 0.03 | 6,4 | 60 | 40 | 0 | 0 |
| *Veronica polita* | 41 | 4 | 4 | 0.06 ± 0.03 | 9,8 | 100 | 0 | 0 | 0 |
| *Euphorbia peplus* | 44 | 3 | 3 | 0.05 ± 0.03 | 6,8 | 100 | 0 | 0 | 0 |
| *Capsella bursa-pastoris* | 51 | 6 | 6 | 0.05 ± 0.02 | 11,8 | 66.7 | 16.7 | 16.7 | 0 |
| *Hypochaeris achyrophorus* | 35 | 4 | 2 | 0.04 ± 0.03 | 11,4 | 0 | 50 | 0 | 50 |
| *Viola arvensis* | 22 | 1 | 1 | 0.03 ± 0.03 | 4,5 | 0 | 0 | 100 | 0 |
| *Bromus diandrus* | 41 | 1 | 1 | 0.02 ± 0.02 | 2,4 | 0 | 0 | 0 | 100 |
| *Papaver rhoeas* | 40 | 1 | 1 | 0.02 ± 0.02 | 2,5 | 0 | 100 | 0 | 0 |
| *Euphorbia helioscopia* | 37 | 1 | 1 | 0.02 ± 0.02 | 2,7 | 0 | 100 | 0 | 0 |
| *Veronica hederifolia* | 27 | 1 | 1 | 0.02 ± 0.02 | 3,7 | 100 | 0 | 0 | 0 |
| *Lysimachia foemina* | 58 | 2 | 1 | 0.01 ± 0.01 | 3,4 | 0 | 100 | 0 | 0 |
| *Senecio vulgaris* | 57 | 1 | 1 | 0.01 ± 0.01 | 1,8 | 100 | 0 | 0 | 0 |
| *Poaceae** | 657 | 203 | 239 | NA | 30,9 | 33.9 | 28.9 | 20.5 | 16.7 |
| *Anacyclus clavatus**** | 2 | 2 | 1 | NA | 100,0 | 0.00 | 0.00 | 0.00 | 100.00 |
| *Anthyllis circinnata**** | 1 | 1 | 1 | NA | 100,0 | 0.00 | 100.00 | 0.00 | 0.00 |
| *Asteraceae**** | 10 | 6 | 31 | NA | 60,0 | 12.90 | 16.13 | 22.58 | 48.39 |
| *Avena fatua**** | 13 | 4 | 6 | NA | 30,8 | 0.00 | 33.33 | 50.00 | 16.67 |
| *Avena spp.**** | 1 | 1 | 1 | NA | 100,0 | 0.00 | 0.00 | 0.00 | 100.00 |
| *Bellardia trixago**** | 15 | 3 | 4 | NA | 20,0 | 0.00 | 25.00 | 75.00 | 0.00 |
| *Bombycilaena sp.**** | 1 | 1 | 2 | NA | 100,0 | 100.00 | 0.00 | 0.00 | 0.00 |
| *Cerastium glomeratum**** | 13 | 3 | 4 | NA | 23,1 | 100.00 | 0.00 | 0.00 | 0.00 |
| *Diplotaxis tenuifolia**** | 3 | 2 | 3 | NA | 66,7 | 0.00 | 0.00 | 0.00 | 100.00 |
| *Echium asperrimum**** | 3 | 1 | 13 | NA | 33,3 | 0.00 | 0.00 | 46.15 | 53.85 |
| *Euphorbia spp.**** | 3 | 1 | 1 | NA | 33,3 | 100.00 | 0.00 | 0.00 | 0.00 |
| *Fallopia convolvulus**** | 1 | 1 | 1 | NA | 100,0 | 0.00 | 100.00 | 0.00 | 0.00 |
| *Galium spp.**** | 6 | 3 | 20 | NA | 50,0 | 5.00 | 10.00 | 15.00 | 70.00 |
| *Glebionis coronaria**** | 7 | 5 | 7 | NA | 71,4 | 71.43 | 14.29 | 14.29 | 0.00 |
| *Hedypnois rhagadioloides**** | 2 | 1 | 3 | NA | 50,0 | 0.00 | 0.00 | 0.00 | 100.00 |
| *Dittrichia viscosa**** | 2 | 1 | 6 | NA | 50,0 | 0.00 | 33.33 | 33.33 | 33.33 |
| *Lactuca virosa**** | 4 | 1 | 1 | NA | 25,0 | 0.00 | 100.00 | 0.00 | 0.00 |
| *Lathyrus aphaca**** | 8 | 3 | 3 | NA | 37,5 | 0.00 | 66.67 | 0.00 | 33.33 |
| *Lathyrus hirsutus**** | 1 | 1 | 1 | NA | 100,0 | 0.00 | 0.00 | 100.00 | 0.00 |
| *Lathyrus ochrus**** | 2 | 1 | 1 | NA | 50,0 | 0.00 | 0.00 | 100.00 | 0.00 |
| *Legousia speculum-veneris**** | 14 | 1 | 3 | NA | 7,1 | 33.33 | 33.33 | 33.33 | 0.00 |
| *Leontodon hispidus**** | 6 | 1 | 3 | NA | 16,7 | 100.00 | 0.00 | 0.00 | 0.00 |
| *Linum strictum**** | 2 | 1 | 1 | NA | 50,0 | 100.00 | 0.00 | 0.00 | 0.00 |
| *Lolium spp.**** | 4 | 1 | 2 | NA | 25,0 | 50.00 | 50.00 | 0.00 | 0.00 |
| *Medicago rugosa**** | 1 | 1 | 1 | NA | 100,0 | 0.00 | 0.00 | 100.00 | 0.00 |
| *Medicago sativa**** | 5 | 2 | 6 | NA | 40,0 | 16.67 | 0.00 | 0.00 | 83.33 |
| *Mentha sp.**** | 5 | 1 | 1 | NA | 20,0 | 0.00 | 100.00 | 0.00 | 0.00 |
| *Olea europaea**** | 5 | 4 | 5 | NA | 80,0 | 0.00 | 40.00 | 0.00 | 60.00 |
| *Ononis viscosa**** | 2 | 1 | 1 | NA | 50,0 | 100.00 | 0.00 | 0.00 | 0.00 |
| *Sanguisorba minor**** | 11 | 7 | 7 | NA | 63,6 | 0.00 | 71.43 | 0.00 | 28.57 |
| *Raphanus raphanistrum**** | 2 | 2 | 3 | NA | 100,0 | 0.00 | 33.33 | 33.33 | 33.33 |
| *Rhagadiolus sp.**** | 2 | 1 | 1 | NA | 50,0 | 0.00 | 100.00 | 0.00 | 0.00 |
| *Seseli sp.**** | 3 | 2 | 10 | NA | 66,7 | 80.00 | 0.00 | 0.00 | 20.00 |
| *Silene sp.**** | 1 | 1 | 4 | NA | 100,0 | 100.00 | 0.00 | 0.00 | 0.00 |
| *Sisymbrium officinale**** | 2 | 2 | 2 | NA | 100,0 | 0.00 | 0.00 | 50.00 | 50.00 |
| *Sium sp.**** | 2 | 1 | 1 | NA | 50,0 | 100.00 | 0.00 | 0.00 | 0.00 |
| *Torilis arvensis**** | 3 | 1 | 1 | NA | 33,3 | 0.00 | 0.00 | 0.00 | 100.00 |
| *Tragopogon porrifolius**** | 4 | 4 | 13 | NA | 100,0 | 53.85 | 38.46 | 0.00 | 7.69 |
| *Trifolium glomeratum**** | 2 | 2 | 2 | NA | 100,0 | 0.00 | 0.00 | 0.00 | 100.00 |
| *Trifolium repens**** | 7 | 1 | 1 | NA | 14,3 | 100.00 | 0.00 | 0.00 | 0.00 |
| *Trifolium spp.**** | 9 | 2 | 6 | NA | 22,2 | 83.33 | 16.67 | 0.00 | 0.00 |
| *Trifolium tomentosum**** | 7 | 1 | 1 | NA | 14,3 | 0.00 | 0.00 | 0.00 | 100.00 |
| *Tripodion tetraphyllum**** | 3 | 1 | 1 | NA | 33,3 | 0.00 | 100.00 | 0.00 | 0.00 |
| *Vicia bithynica**** | 2 | 1 | 1 | NA | 50,0 | 0.00 | 0.00 | 0.00 | 100.00 |
| *Vicia Lathyroides**** | 2 | 2 | 3 | NA | 100,0 | 33.33 | 66.67 | 0.00 | 0.00 |
| *Achillea millefolium*** | 1 | 0 | 0 | NA | 0,0 | NA | NA | NA | NA |
| *Adonis annua*** | 8 | 0 | 0 | NA | 0,0 | NA | NA | NA | NA |
| *Ajuga chamaepitys*** | 5 | 0 | 0 | NA | 0,0 | NA | NA | NA | NA |
| *Allium neapolitanum*** | 3 | 0 | 0 | NA | 0,0 | NA | NA | NA | NA |
| *Allium roseum*** | 1 | 0 | 0 | NA | 0,0 | NA | NA | NA | NA |
| *Allium* spp.** | 1 | 0 | 0 | NA | 0,0 | NA | NA | NA | NA |
| *Anemone coronaria*** | 2 | 0 | 0 | NA | 0,0 | NA | NA | NA | NA |
| *Anthyllis vulneraria*** | 1 | 0 | 0 | NA | 0,0 | NA | NA | NA | NA |
| *Arisarum vulgare*** | 3 | 0 | 0 | NA | 0,0 | NA | NA | NA | NA |
| *Artemisia* sp.** | 4 | 0 | 0 | NA | 0,0 | NA | NA | NA | NA |
| *Asparagus officinalis*** | 1 | 0 | 0 | NA | 0,0 | NA | NA | NA | NA |
| *Astragalus hamosus*** | 66 | 0 | 0 | NA | 0,0 | NA | NA | NA | NA |
| *Blackstonia perfoliata*** | 1 | 0 | 0 | NA | 0,0 | NA | NA | NA | NA |
| *Bromus hordeaceus*** | 3 | 0 | 0 | NA | 0,0 | NA | NA | NA | NA |
| *Buglossoides arvensis*** | 4 | 0 | 0 | NA | 0 | NA | NA | NA | NA |
| *Campanula* sp.** | 2 | 0 | 0 | NA | 0 | NA | NA | NA | NA |
| *Cardamine hirsute*** | 1 | 0 | 0 | NA | 0 | NA | NA | NA | NA |
| *Chenopodium album*** | 7 | 0 | 0 | NA | 0 | NA | NA | NA | NA |
| *Cirsium acaule*** | 1 | 0 | 0 | NA | 0 | NA | NA | NA | NA |
| *Cirsium arvense*** | 1 | 0 | 0 | NA | 0 | NA | NA | NA | NA |
| *Convolvulus althaeoides*** | 7 | 0 | 0 | NA | 0 | NA | NA | NA | NA |
| *Convolvulus* sp.** | 4 | 0 | 0 | NA | 0 | NA | NA | NA | NA |
| *Cynodon dactilon*** | 1 | 0 | 0 | NA | 0 | NA | NA | NA | NA |
| *Cynoglossum creticum*** | 1 | 0 | 0 | NA | 0 | NA | NA | NA | NA |
| *Dasypirum villosum*** | 1 | 0 | 0 | NA | 0 | NA | NA | NA | NA |
| *Echium plantagineum*** | 1 | 0 | 0 | NA | 0 | NA | NA | NA | NA |
| *Euphorbia exigua*** | 1 | 0 | 0 | NA | 0 | NA | NA | NA | NA |
| *Fedia graciliflora*** | 13 | 0 | 0 | NA | 0 | NA | NA | NA | NA |
| *Ficaria verna*** | 8 | 0 | 0 | NA | 0 | NA | NA | NA | NA |
| *Filago* sp.** | 1 | 0 | 0 | NA | 0 | NA | NA | NA | NA |
| *Fumaria officinalis*** | 6 | 0 | 0 | NA | 0 | NA | NA | NA | NA |
| *Galactites tomentosum*** | 1 | 0 | 0 | NA | 0 | NA | NA | NA | NA |
| *Geranium molle*** | 1 | 0 | 0 | NA | 0 | NA | NA | NA | NA |
| *Geranium purpureum*** | 2 | 0 | 0 | NA | 0 | NA | NA | NA | NA |
| *Hordeum murinum*** | 6 | 0 | 0 | NA | 0 | NA | NA | NA | NA |
| *Hypericum perforatum*** | 1 | 0 | 0 | NA | 0 | NA | NA | NA | NA |
| *Iris bulbosa*** | 1 | 0 | 0 | NA | 0 | NA | NA | NA | NA |
| *Lappula occidentalis*** | 1 | 0 | 0 | NA | 0 | NA | NA | NA | NA |
| *Lathyrus annuus*** | 2 | 0 | 0 | NA | 0 | NA | NA | NA | NA |
| *Lathyrus latifolius*** | 5 | 0 | 0 | NA | 0 | NA | NA | NA | NA |
| *Lathyrus pratensis*** | 1 | 0 | 0 | NA | 0 | NA | NA | NA | NA |
| *Leugosia hybrida*** | 1 | 0 | 0 | NA | 0 | NA | NA | NA | NA |
| *Leugosia* sp.** | 1 | 0 | 0 | NA | 0 | NA | NA | NA | NA |
| *Linaria reflexa*** | 6 | 0 | 0 | NA | 0 | NA | NA | NA | NA |
| *Linaria* sp.** | 1 | 0 | 0 | NA | 0 | NA | NA | NA | NA |
| *Linum* sp.** | 8 | 0 | 0 | NA | 0 | NA | NA | NA | NA |
| *Lotus* sp.** | 1 | 0 | 0 | NA | 0 | NA | NA | NA | NA |
| *Lysimachia arvensis*** | 2 | 0 | 0 | NA | 0 | NA | NA | NA | NA |
| *Lysimachia spp.*** | 7 | 0 | 0 | NA | 0 | NA | NA | NA | NA |
| *Medicago* sp.** | 1 | 0 | 0 | NA | 0 | NA | NA | NA | NA |
| *Melissa officinalis*** | 3 | 0 | 0 | NA | 0 | NA | NA | NA | NA |
| *Myosotis* sp.** | 2 | 0 | 0 | NA | 0 | NA | NA | NA | NA |
| *Onobrychis caput-gallI*** | 1 | 0 | 0 | NA | 0 | NA | NA | NA | NA |
| *Ornithogalum umbellatum*** | 3 | 0 | 0 | NA | 0 | NA | NA | NA | NA |
| *Oxalis pes-caprae*** | 21 | 0 | 0 | NA | 0 | NA | NA | NA | NA |
| *Pilosella* sp.** | 3 | 0 | 0 | NA | 0 | NA | NA | NA | NA |
| *Plantago lanceolata*** | 11 | 0 | 0 | NA | 0 | NA | NA | NA | NA |
| *Poa annua*** | 14 | 0 | 0 | NA | 0 | NA | NA | NA | NA |
| *Polygala monspeliaca*** | 1 | 0 | 0 | NA | 0 | NA | NA | NA | NA |
| *Reseda alba*** | 1 | 0 | 0 | NA | 0 | NA | NA | NA | NA |
| *Reseda lutea*** | 2 | 0 | 0 | NA | 0 | NA | NA | NA | NA |
| *Rumex acetosa*** | 7 | 0 | 0 | NA | 0 | NA | NA | NA | NA |
| *Rumex bucephalophorus*** | 2 | 0 | 0 | NA | 0 | NA | NA | NA | NA |
| *Sedum* sp.** | 7 | 0 | 0 | NA | 0 | NA | NA | NA | NA |
| *Thlaspi arvensis*** | 4 | 0 | 0 | NA | 0 | NA | NA | NA | NA |
| *Trifolium campestre*** | 3 | 0 | 0 | NA | 0 | NA | NA | NA | NA |
| *Trifolium pratense*** | 9 | 0 | 0 | NA | 0 | NA | NA | NA | NA |
| *Triticum* spp.** | 4 | 0 | 0 | NA | 0 | NA | NA | NA | NA |
| *Triticum vulgare*** | 1 | 0 | 0 | NA | 0 | NA | NA | NA | NA |
| *Tyrimnus leucographus*** | 2 | 0 | 0 | NA | 0 | NA | NA | NA | NA |
| *Tyrimnus* sp.** | 1 | 0 | 0 | NA | 0 | NA | NA | NA | NA |
| *Urtica dioica*** | 2 | 0 | 0 | NA | 0 | NA | NA | NA | NA |
| *Verbascum sinuatum*** | 4 | 0 | 0 | NA | 0 | NA | NA | NA | NA |
| *Veronica arvensis*** | 7 | 0 | 0 | NA | 0 | NA | NA | NA | NA |
| *Veronica persica*** | 5 | 0 | 0 | NA | 0 | NA | NA | NA | NA |
| *Veronica* sp.** | 5 | 0 | 0 | NA | 0 | NA | NA | NA | NA |
| *Vicia cracca*** | 1 | 0 | 0 | NA | 0 | NA | NA | NA | NA |
| *Vicia lutea*** | 9 | 0 | 0 | NA | 0 | NA | NA | NA | NA |
| *Vicia* spp.** | 5 | 0 | 0 | NA | 0 | NA | NA | NA | NA |
| *Vicia villosa*** | 2 | 0 | 0 | NA | 0 | NA | NA | NA | NA |
| *Vitis* sp.** | 2 | 0 | 0 | NA | 0 | NA | NA | NA | NA |
| *Transect species identified to family level indicates pooled records of specimens that could not be identified below family level and do not represent a single botanical species, **unsuitable plant species, ***Hostplant species occurred < 15 times in the entire survey, are not included in statistical analysis. | | | | | | | | | |

**Table S2**. List of host plant species (ranked by estimated nymph abundance) and non-suitable species of N. campestris nymphs. Mean estimates ± standard errors derived from generalized linear mixed models are shown.

|  | **Plant occurrences in transects** | **Occurrences of *N. campestris*** | **Nymph abundance** | **Mean ± SE** | **Occurrence rate** | **1–2nd instar %** | **3rd instar %** | **4th instar %** | **5th instar %** |
| --- | --- | --- | --- | --- | --- | --- | --- | --- | --- |
| **Transect species** |  | | | | | | | | |
| *Avena barbata* | 24 | 16 | 25 | 0.50 ± 0.34 | 66,7 | 0 | 8 | 60 | 32 |
| *Bromus diandrus* | 64 | 30 | 33 | 0.36 ± 0.16 | 46,9 | 15.2 | 3 | 33.3 | 48.5 |
| *Hordeum vulgare* | 58 | 8 | 18 | 0.14 ± 0.08 | 13,8 | 38.9 | 33.3 | 22.2 | 5.6 |
| *Lolium perenne* | 58 | 9 | 9 | 0.08 ± 0.05 | 15,5 | 11 | 0 | 44.5 | 44.5 |
| *Avena fatua* | 30 | 2 | 2 | 0.04 ± 0.04 | 6,7 | 0 | 100 | 0 | 0 |
| *Crepis spp.* | 36 | 1 | 2 | 0.02 ± 0.02 | 2,8 | 100 | 0 | 0 | 0 |
| *Sonchus asper* | 57 | 1 | 4 | 0.02 ± 0.02 | 1,8 | 100 | 0 | 0 | 0 |
| *Galium mollugo* | 53 | 1 | 2 | 0.01 ± 0.01 | 1,9 | 100 | 0 | 0 | 0 |
| *Sonchus oleraceus* | 371 | 2 | 5 | 0.01 ± 0.00 | 0,5 | 100 | 0 | 0 | 0 |
| *Erodium cicutarium* | 108 | 1 | 1 | 0.01 ± 0.01 | 0,9 | 100 | 0 | 0 | 0 |
| *Calendula arvensis* | 456 | 2 | 2 | 0.00 ± 0.00 | 0,4 | 100 | 0 | 0 | 100 |
| *Daucus carota* | 235 | 1 | 1 | 0.00 ± 0.00 | 0,4 | 0 | 0 | 0 | 100 |
| *Medicago truncatula* | 255 | 1 | 1 | 0.00 ± 0.00 | 0,4 | 0 | 100 | 0 | 0 |
| *Sherardia arvensis)* | 299 | 1 | 1 | 0.00 ± 0.00 | 0,3 | 0 | 100 | 0 | 0 |
| *Poaceae** | 877 | 829 | 1046 | NA | 94,5 | 45.4 | 25.1 | 21.9 | 7.6 |
| *Adonis annua*** | 17 | 0 | 0 | NA | 0,0 | 0 | 0 | 0 | 0 |
| *Anthemis arvensis*** | 63 | 0 | 0 | NA | 0,0 | 0 | 0 | 0 | 0 |
| *Astragalus hamosus*** | 90 | 0 | 0 | NA | 0,0 | 0 | 0 | 0 | 0 |
| *Capsella bursa-pastoris*** | 44 | 0 | 0 | NA | 0,0 | 0 | 0 | 0 | 0 |
| *Carduus pycnocephalus*** | 22 | 0 | 0 | NA | 0,0 | 0 | 0 | 0 | 0 |
| *Chenopodium album*** | 18 | 0 | 0 | NA | 0,0 | 0 | 0 | 0 | 0 |
| *Cichorium inthybus*** | 21 | 0 | 0 | NA | 0,0 | 0 | 0 | 0 | 0 |
| *Convolvulus arvensis*** | 148 | 0 | 0 | NA | 0,0 | 0 | 0 | 0 | 0 |
| *Coronilla scorpioides*** | 41 | 0 | 0 | NA | 0,0 | 0 | 0 | 0 | 0 |
| *Crepis bursifolia*** | 87 | 0 | 0 | NA | 0,0 | 0 | 0 | 0 | 0 |
| *Crepis sancta*** | 228 | 0 | 0 | NA | 0,0 | 0 | 0 | 0 | 0 |
| *Crepis vesicaria*** | 45 | 0 | 0 | NA | 0,0 | 0 | 0 | 0 | 0 |
| *Diplotaxis erucoides*** | 66 | 0 | 0 | NA | 0,0 | 0 | 0 | 0 | 0 |
| *Erigeron canadensis*** | 28 | 0 | 0 | NA | 0,0 | 0 | 0 | 0 | 0 |
| *Erodium malacoides*** | 158 | 0 | 0 | NA | 0,0 | 0 | 0 | 0 | 0 |
| *Euphorbia helioscopia*** | 90 | 0 | 0 | NA | 0,0 | 0 | 0 | 0 | 0 |
| *Euphorbia peplus*** | 68 | 0 | 0 | NA | 0,0 | 0 | 0 | 0 | 0 |
| *Fedia graciliflora*** | 51 | 0 | 0 | NA | 0,0 | 0 | 0 | 0 | 0 |
| *Foeniculum vulgare*** | 24 | 0 | 0 | NA | 0,0 | 0 | 0 | 0 | 0 |
| *Galium aparine*** | 32 | 0 | 0 | NA | 0,0 | 0 | 0 | 0 | 0 |
| *Geranium rotundifolium*** | 114 | 0 | 0 | NA | 0,0 | 0 | 0 | 0 | 0 |
| *Glebionis segetum*** | 100 | 0 | 0 | NA | 0,0 | 0 | 0 | 0 | 0 |
| *Hedypnois rhagadioloides*** | 19 | 0 | 0 | NA | 0,0 | 0 | 0 | 0 | 0 |
| *Hyoseris radiata*** | 44 | 0 | 0 | NA | 0,0 | 0 | 0 | 0 | 0 |
| *Hypochaeris achyrophorus*** | 38 | 0 | 0 | NA | 0,0 | 0 | 0 | 0 | 0 |
| *Lamium amplexicaule*** | 40 | 0 | 0 | NA | 0,0 | 0 | 0 | 0 | 0 |
| *Leugosia speculum-veneris*** | 20 | 0 | 0 | NA | 0,0 | 0 | 0 | 0 | 0 |
| *Linaria reflexa*** | 18 | 0 | 0 | NA | 0,0 | 0 | 0 | 0 | 0 |
| *Lotus ornithopodioides*** | 55 | 0 | 0 | NA | 0,0 | 0 | 0 | 0 | 0 |
| *Lysimachia foemina*** | 79 | 0 | 0 | NA | 0,0 | 0 | 0 | 0 | 0 |
| *Malva sylvestris*** | 55 | 0 | 0 | NA | 0,0 | 0 | 0 | 0 | 0 |
| *Matricaria chamomilla*** | 36 | 0 | 0 | NA | 0,0 | 0 | 0 | 0 | 0 |
| *Medicago arabica*** | 225 | 0 | 0 | NA | 0,0 | 0 | 0 | 0 | 0 |
| *Medicago minima*** | 33 | 0 | 0 | NA | 0,0 | 0 | 0 | 0 | 0 |
| *Medicago orbicularis*** | 131 | 0 | 0 | NA | 0,0 | 0 | 0 | 0 | 0 |
| *Medicago polymorpha*** | 206 | 0 | 0 | NA | 0,0 | 0 | 0 | 0 | 0 |
| *Melilotus sulcatus*** | 18 | 0 | 0 | NA | 0,0 | 0 | 0 | 0 | 0 |
| *Mercurialis annua*** | 48 | 0 | 0 | NA | 0,0 | 0 | 0 | 0 | 0 |
| *Muscari latifolium*** | 48 | 0 | 0 | NA | 0,0 | 0 | 0 | 0 | 0 |
| *Onobrychis viciifolia*** | 31 | 0 | 0 | NA | 0,0 | 0 | 0 | 0 | 0 |
| *Oxalis pes-caprae*** | 25 | 0 | 0 | NA | 0,0 | 0 | 0 | 0 | 0 |
| *Papaver rhoaes*** | 53 | 0 | 0 | NA | 0,0 | 0 | 0 | 0 | 0 |
| *Picris hieracioides*** | 54 | 0 | 0 | NA | 0,0 | 0 | 0 | 0 | 0 |
| *Plantago afra*** | 36 | 0 | 0 | NA | 0,0 | 0 | 0 | 0 | 0 |
| *Plantago psilli*** | 21 | 0 | 0 | NA | 0,0 | 0 | 0 | 0 | 0 |
| *Reichardia picroides*** | 39 | 0 | 0 | NA | 0,0 | 0 | 0 | 0 | 0 |
| *Salvia verbenaca*** | 31 | 0 | 0 | NA | 0,0 | 0 | 0 | 0 | 0 |
| *Scandix pecten-veneris*** | 199 | 0 | 0 | NA | 0,0 | 0 | 0 | 0 | 0 |
| *Scorpiurus muricatus*** | 27 | 0 | 0 | NA | 0,0 | 0 | 0 | 0 | 0 |
| *Senecio vulgaris*** | 49 | 0 | 0 | NA | 0,0 | 0 | 0 | 0 | 0 |
| *Sonchus tenerrimus*** | 32 | 0 | 0 | NA | 0,0 | 0 | 0 | 0 | 0 |
| *Stellaria media*** | 160 | 0 | 0 | NA | 0,0 | 0 | 0 | 0 | 0 |
| *Taraxacum officinale*** | 23 | 0 | 0 | NA | 0,0 | 0 | 0 | 0 | 0 |
| *Tordylium apulum*** | 83 | 0 | 0 | NA | 0,0 | 0 | 0 | 0 | 0 |
| *Trifolium stellatum*** | 95 | 0 | 0 | NA | 0,0 | 0 | 0 | 0 | 0 |
| *Trifolium tomentosum*** | 18 | 0 | 0 | NA | 0,0 | 0 | 0 | 0 | 0 |
| *Urospermum picroides*** | 113 | 0 | 0 | NA | 0,0 | 0 | 0 | 0 | 0 |
| *Valerianella locusta*** | 64 | 0 | 0 | NA | 0,0 | 0 | 0 | 0 | 0 |
| *Veronica hederifolia*** | 32 | 0 | 0 | NA | 0,0 | 0 | 0 | 0 | 0 |
| *Veronica polita*** | 62 | 0 | 0 | NA | 0,0 | 0 | 0 | 0 | 0 |
| *Vicia sativa*** | 115 | 0 | 0 | NA | 0,0 | 0 | 0 | 0 | 0 |
| *Viola arvensis*** | 34 | 0 | 0 | NA | 0,0 | 0 | 0 | 0 | 0 |
| *Transect species identified to family level indicates pooled records of specimens that could not be identified below family level and do not represent a single botanical species, **unsuitable plant species, ***Hostplant species occurred < 15 times in the entire survey are not included in statistical analysis. | | | | | | | | | |

**Table S3.** Instar-specific *P. spumarius* estimated nymph abundance on host plant species. Mean estimates ± standard errors derived from generalized linear mixed models are shown.

| **Transect species** | **1-2^nd^ instar (Mean ± SE)** | **3^rd^ instar (Mean ± SE)** | **4^th^ instar (Mean ± SE)** | **5^th^ instar (Mean ± SE)** |
| --- | --- | --- | --- | --- |
| *Anthemis arvensis* | 0.008 ± 0.009 | 0.034 ± 0.021 | 0.034 ± 0.018 | 0.028 ± 0.014 |
| *Avena barbata* | NA | NA | 0.029 ± 0.031 | 0.055 ± 0.034 |
| *Bellardia trixago* | NA | 0.031 ± NA | 0.057 ± 0.043 | NA |
| *Bromus diandrus* | NA | NA | NA | 0.004 ± 0.004 |
| *Calendula arvensis* | 0.087 ± 0.018 | 0.090 ± NA | 0.058 ± 0.013 | 0.019 ± 0.006 |
| *Capsella bursa-pastoris* | 0.012 ± 0.007 | 0.006 ± NA | 0.006 ± 0.007 | NA |
| *Carduus pycnocephalus* | NA | NA | NA | 0.053 ± 0.032 |
| *Cichorium inthybus* | NA | NA | 0.006 ± 0.007 | 0.076 ± 0.046 |
| *Chondrilla juncea* | 0.029 ± 0.019 | NA | NA | NA |
| *Convolvulus arvensis* | 0.037 ± 0.013 | 0.023 ± NA | NA | 0.003 ± 0.003 |
| *Coronilla scorpioides* | 0.027 ± 0.021 | 0.028 ± NA | NA | 0.018 ± 0.015 |
| *Crepis bursifolia* | 0.085 ± 0.025 | NA | 0.007 ± 0.005 | 0.008 ± 0.005 |
| *Crepis sancta* | 0.052 ± 0.014 | 0.003 ± NA | 0.003 ± 0.003 | NA |
| *Crepis spp.* | 0.125 ± 0.050 | 0.067 ± NA | NA | NA |
| *Crepis vesicaria* | 0.152 ± 0.045 | 0.051 ± NA | 0.024 ± 0.014 | 0.019 ± 0.013 |
| *Daucus carota* | 0.058 ± 0.016 | 0.072 ± NA | 0.040 ± 0.013 | 0.038 ± 0.013 |
| *Diplotaxis erucoides* | 0.026 ± 0.014 | NA | NA | 0.007 ± 0.007 |
| *Erigeron canadensis* | 0.208 ± 0.073 | 0.095 ± NA | 0.032 ± 0.020 | 0.122 ± 0.052 |
| *Erodium cicutarium* | 0.052 ± 0.017 | 0.049 ± 0.018 | 0.049 ± 0.019 | 0.018 ± 0.010 |
| *Erodium malacoides* | 0.045 ± 0.013 | 0.054 ± 0.016 | 0.039 ± 0.014 | 0.034 ± 0.012 |
| *Euphorbia helioscopia* | NA | 0.011 ± 0.012 | NA | NA |
| *Euphorbia peplus* | 0.018 ± 0.011 | NA | NA | NA |
| *Foeniculum vulgare* | 0.463 ± 0.214 | 0.142 ± 0.070 | 0.217 ± 0.107 | 0.008 ± 0.008 |
| *Galium aparine* | 0.048 ± 0.027 | 0.045 ± 0.031 | 0.046 ± 0.031 | 0.011 ± 0.012 |
| *Galium mollugo* | 0.024 ± 0.014 | 0.013 ± 0.014 | NA | NA |
| *Geranium rotundifolium* | 0.029 ± 0.012 | NA | NA | NA |
| *Glebionis segetum* | 0.024 ± 0.014 | 0.055 ± 0.024 | NA | NA |
| *Hordeum vulgare* | NA | 0.033 ± 0.026 | 0.044 ± 0.030 | 0.018 ± 0.014 |
| *Hyoseris radiata* | 0.025 ± 0.028 | 0.026 ± 0.028 | 0.018 ± 0.019 | NA |
| *Hypochaeris achyrophorus* | NA | 0.018 ± 0.019 | NA | 0.003 ± 0.003 |
| *Lamium amplexicaule* | 0.011 ± 0.007 | 0.016 ± 0.013 | NA | NA |
| *Lolium perenne* | 0.042 ± 0.023 | 0.020 ± 0.014 | 0.051 ± 0.024 | 0.022 ± 0.012 |
| *Lotus ornithopodioides* | 0.013 ± 0.013 | 0.022 ± 0.017 | 0.012 ± 0.013 | 0.008 ± 0.008 |
| *Lysimachia foemina* | NA | 0.007 ± 0.008 | NA | NA |
| *Malva sylvestris* | 0.069 ± 0.025 | 0.058 ± 0.024 | 0.033 ± 0.017 | 0.077 ± 0.030 |
| *Matricaria chamomilla* | 0.042 ± 0.037 | 0.072 ± 0.052 | NA | 0.010 ± 0.011 |
| *Medicago arabica* | 0.069 ± 0.016 | 0.073 ± 0.018 | 0.018 ± 0.007 | 0.007 ± 0.004 |
| *Medicago minima* | NA | 0.013 ± 0.014 | 0.014 ± 0.015 | 0.007 ± 0.008 |
| *Medicago orbicularis* | NA | 0.053 ± 0.022 | 0.018 ± 0.010 | 0.016 ± 0.007 |
| *Medicago polymorpha* | 0.063 ± 0.017 | 0.060 ± 0.017 | 0.046 ± 0.014 | 0.048 ± 0.015 |
| *Medicago truncatula* | 0.030 ± 0.009 | 0.040 ± 0.011 | 0.027 ± 0.009 | 0.023 ± 0.008 |
| *Melilotus sulcatus* | 0.032 ± 0.020 | 0.022 ± 0.023 | 0.050 ± 0.040 | 0.045 ± 0.032 |
| *Onobrychis viciifolia* | 0.011 ± 0.012 | 0.032 ± 0.025 | 0.069 ± 0.041 | 0.030 ± 0.019 |
| *Papaver rhoeas* | NA | 0.011 ± 0.012 | NA | NA |
| *Picris hieracioides* | 0.014 ± 0.011 | 0.053 ± 0.027 | 0.008 ± 0.008 | 0.019 ± 0.013 |
| *Plantago afra* | 0.012 ± 0.013 | 0.013 ± 0.013 | 0.062 ± 0.037 | 0.017 ± 0.010 |
| *Reichardia picroides* | 0.088 ± 0.040 | 0.083 ± 0.046 | 0.013 ± 0.014 | 0.014 ± 0.015 |
| *Salvia verbenaca* | 0.075 ± 0.040 | 0.015 ± 0.016 | 0.036 ± 0.027 | 0.101 ± 0.052 |
| *Scabiosa atropurpurea* | 0.019 ± 0.020 | 0.016 ± 0.010 | NA | 0.014 ± 0.015 |
| *Scandix pecten-veneris* | 0.047 ± 0.015 | NA | NA | 0.007 ± 0.005 |
| *Scorpiurus muricatus* | NA | 0.016 ± 0.017 | NA | 0.012 ± 0.009 |
| *Senecio vulgaris* | 0.004 ± 0.004 | NA | NA | NA |
| *Sherardia arvensis* | 0.022 ± 0.007 | 0.018 ± 0.007 | NA | NA |
| *Sonchus asper* | 0.149 ± 0.046 | 0.193 ± 0.057 | 0.329 ± 0.093 | 0.092 ± 0.036 |
| *Sonchus oleraceus* | 0.020 ± 0.006 | 0.031 ± 0.009 | 0.030 ± 0.009 | 0.042 ± 0.012 |
| *Sonchus tenerrimus* | 0.075 ± 0.029 | 0.033 ± 0.019 | 0.023 ± 0.015 | 0.054 ± 0.025 |
| *Stellaria media* | 0.027 ± 0.009 | NA | NA | 0.010 ± 0.006 |
| *Taraxacum officinale* | NA | 0.098 ± 0.055 | 0.071 ± 0.050 | 0.028 ± 0.031 |
| *Tordylium apulum* | 0.026 ± 0.014 | NA | 0.010 ± 0.011 | NA |
| *Trifolium stellatum* | 0.013 ± 0.013 | 0.010 ± 0.010 | 0.006 ± 0.006 | 0.006 ± 0.006 |
| *Urospermum dalechampii* | 0.193 ± 0.129 | 0.084 ± 0.066 | 0.081 ± 0.061 | 0.190 ± 0.108 |
| *Urospermum picroides* | 0.024 ± 0.013 | 0.094 ± 0.031 | 0.049 ± 0.019 | 0.011 ± 0.007 |
| *Valerianella locusta* | 0.040 ± 0.022 | NA | NA | 0.015 ± 0.012 |
| *Veronica hederifolia* | 0.008 ± 0.009 | NA | NA | NA |
| *Veronica polita* | 0.016 ± 0.009 | NA | NA | NA |
| *Vicia sativa* | 0.040 ± 0.018 | 0.034 ± 0.017 | 0.026 ± 0.013 | 0.021 ± 0.011 |
| *Viola arvensis* | NA | NA | 0.029 ± 0.031 | NA |

**Table S4.** List of host plant families (ranked by plant occurrences in transects) of P. spumarius nymphs.

| **Transect plant families** | **Plant occurrences in transects** | **Occurrences of P. spumarius** | **Occurrence rate %** |
| --- | --- | --- | --- |
| Asteraceae | 1660 | 602 | 36,3 |
| Fabaceae | 1131 | 335 | 29,6 |
| Poaceae | 845 | 245 | 29,0 |
| Apiaceae | 344 | 95 | 27,6 |
| Geraniaceae | 328 | 83 | 25,3 |
| Rubiaceae | 290 | 39 | 13,4 |
| Plantaginaceae | 146 | 18 | 12,3 |
| Caryophyllaceae | 135 | 21 | 15,6 |
| Brassicaceae | 111 | 16 | 14,4 |
| Euphorbiaceae | 111 | 5 | 4,5 |
| Convolvulaceae | 104 | 17 | 16,3 |
| Lamiaceae | 89 | 11 | 12,4 |
| Primulaceae | 67 | 2 | 3,0 |
| Caprifoliaceae | 64 | 8 | 12,5 |
| Papaveraceae | 62 | 1 | 1,6 |
| Malvaceae | 61 | 23 | 37,7 |
| Liliaceae | 60 | 0 | 0,0 |
| Violaceae | 22 | 1 | 4,5 |
| Oxalidaceae | 21 | 0 | 0,0 |
| Orobanchaceae | 19 | 4 | 21,1 |
| Campanulaceae | 18 | 3 | 16,7 |
| Ranunculaceae | 18 | 0 | 0,0 |
| Rosaceae | 11 | 7 | 63,6 |
| Linaceae | 10 | 1 | 10,0 |
| Polygonaceae | 10 | 1 | 10,0 |
| Boraginaceae | 8 | 0 | 0,0 |
| Amaranthaceae | 7 | 0 | 0,0 |
| Crassulaceae | 7 | 0 | 0,0 |
| Oleaceae | 5 | 4 | 80,0 |
| Araceae | 3 | 0 | 0,0 |
| Resedaceae | 3 | 0 | 0,0 |
| Urticaceae | 2 | 0 | 0,0 |
| Vitaceae | 2 | 0 | 0,0 |
| Asparagaceae | 1 | 0 | 0,0 |
| Gentianaceae | 1 | 0 | 0,0 |
| Hypericaceae | 1 | 0 | 0,0 |
| Iridaceae | 1 | 0 | 0,0 |
| Polygalaceae | 1 | 0 | 0,0 |
